# Dyadic discrimination and mental well-being among older Americans

**DOI:** 10.1093/geroni/igag100

**Published:** 2026-09-04

**Authors:** Jeffrey E Stokes, Heather R Farmer

**Affiliations:** Department of Gerontology, University of Massachusetts Boston, Boston, Massachusetts, United States; Department of Human Development and Family Sciences, University of Delaware, Newark, Delaware, United States

**Keywords:** Mental health, Social determinants of health, Stress & coping, Longitudinal design/data analysis, Structural equation or path model

## Abstract

**Background and Objectives:**

There is a thorough literature on the mental health consequences of discrimination exposure in adulthood, yet little work to date has situated such experiences within a relational, dyadic context. The present brief report analyzes 2-wave longitudinal data from the Health and Retirement Study (2018–2022) to determine whether individuals’ own or their intimate partner’s experiences of discrimination were associated with changes to anxiety symptoms, perceived stress, and/or self-esteem over a 4-year period.

**Research Methods and Design:**

Dyadic lagged dependent variable models were estimated using a structural equation modeling framework with full information maximum likelihood methods to address missing data. A total of 1,484 individuals from 742 opposite-gender couples participated at both the 2018 and 2022 waves of data collection and comprised the analytic sample for this study.

**Results:**

Individuals’ own exposure to everyday discrimination was associated with increased symptoms of anxiety and perceived stress, as well as with reduced self-esteem, over the 4-year period. A dyadic partner’s exposure to everyday discrimination was associated with increased anxiety symptoms for men only, though post-hoc Wald tests failed to find a significant gender difference in effects.

**Discussion and Implications:**

Discrimination is a chronic psychosocial stressor with harmful consequences for mental health of affected individuals even in mid and later life. Moreover, limited evidence suggests that discrimination may have dyadic consequences as well, though these patterns are mixed. Future research is needed to determine interpersonal mechanisms for the findings of interest.

Innovation and Translational Significance: This study highlights everyday discrimination as a couple-level stressor with consequences for both partners’ mental health and well-being within intimate relationships. Findings, however, were mixed, and suggest that the need for couple-level interventions may be limited to specific aspects of mental and emotional health (e.g., anxiety) rather than others (e.g., self-esteem). Further, future work is needed to identify dyadic coping mechanisms that may reduce harmful effects of discrimination on mental health among married/partnered older persons, who appear negatively affected by perceived discrimination despite the social support resources at their disposal.

Self-reported everyday discrimination (hereafter, discrimination)—or experiences of inferior treatment in interpersonal interactions—is a psychosocial stressor that affects individuals throughout the life course (Ayalon & Gum, 2011; Kessler et al., 1999; Mouzon et al., 2017; Williams et al., 1997). With advancing age, the perceived experience of discrimination becomes less frequent, as individuals leave the labor force, exhibit better emotional regulation, and trim their social networks to focus on their closest and most rewarding relationships (Carstensen et al., 1999; Charles, 2010; Kessler et al., 1999). Yet older adults have heightened *vulnerability* to discrimination when it does occur (Charles, 2010). Robust scholarship reveals that discrimination contributes to worse mental and emotional well-being, including in mid- and later life (e.g., Earnshaw et al., 2016; Farmer et al., 2026; Kessler et al., 1999). Specifically, perceived everyday discrimination has been linked with greater stress (Leger et al., 2022), more anxiety (Wang & Narcisse, 2025), and lower self-esteem (Stokes, 2019). Little research, however, has situated experiences of discrimination within midlife and older adults’ *relationship* contexts.

The experience of everyday discrimination is impactful for mental health and well-being in part because it communicates to individuals that they *do not belong* or *are not welcome* within certain social spaces and communities; this can undermine self-esteem and lead to greater anxiety, stress, depression, and social disconnection (Leger et al., 2022; Stokes, 2019, 2020; Wang & Narcisse, 2025). Critically, however, although *exposure to* unfair treatment may be an individual experience, the *consequences* of experiencing discrimination may not be limited to individuals themselves, as the Dyadic Biobehavioral Stress Model reveals (Shrout, 2021). Among married and partnered adults, psychosocial stressors—and the responses they provoke—can be *shared* with intimate partners. Vicarious discrimination occurs when close social partners’ experience of discrimination *indirectly* affects individuals who did not experience discrimination themselves (Louie & Upenieks, 2022). The reactions of the partner exposed to everyday discrimination (e.g., social withdrawal, negative interactions, and interpersonal behaviors) may further lead to *spillover effects* for their closest tie(s), such as inducing stress and anxiety (e.g., Stokes, 2017). For instance, anxiety can be “contagious” within older marriages, such that having an anxious partner is a risk factor for experiencing anxiety oneself (Stokes, 2017). If perceived exposure to discrimination leads to increased anxiety for one partner, this may “spill over” and create heightened levels of anxiety for *both partners* over time.

Yet scholarship on these shared processes within older couples is limited, as extant work emphasizes individual-level exposures rather than interpersonal ones. While some cross-sectional evidence suggests that an intimate partner’s discrimination exposure may be associated with *their partner’s* health and well-being, these associations have not been assessed longitudinally (Wofford et al., 2019). Longitudinal investigations are critical to understand whether associations hold over time or may instead be spurious and/or due to homogamy and selection effects.

This brief report uses longitudinal data from the Health and Retirement Study (HRS) (2018–2022), a nationally representative panel study of adults 51+, to examine whether discrimination is associated with changes to individuals’ own as well as their intimate partners’ anxiety symptoms, perceived stress, and/or self-esteem over 4 years. Findings will contribute to a growing literature on discrimination and mental health science among older couples.

## Method

### Data and sample

Data for this study were drawn from the 2018 and 2022 waves of the HRS, a nationally representative panel study of adults over age 50 in the United States (Sonnega et al., 2014). The HRS sample includes both age-eligible focal participants and any coresident partners/spouses, irrespective of age, thereby providing a dyadic sample of older couples in the United States as well. The HRS administers core surveys every 2 years, while detailed information on psychosocial measures—including everyday discrimination—is routinely collected every 4 years in a Leave Behind Questionnaire (LBQ). There have been changes in the psychological measures included in the LBQ, making longitudinal investigations on these outcomes challenging; for example, information on anxiety symptoms was collected in 2018 for the first time since 2012. As the HRS has grown, it has introduced additional validated measures, including those on perceived stress and self-esteem to the LBQ in 2018. Therefore, the 2018 wave served as the baseline for this study, with 2022 LBQ data used as the follow-up wave.

A total of 1,417 opposite-sex couples had valid dyadic LBQ data in 2018. The final analytic sample included 742 opposite-gender couples (52.36%) who (1) remained intact (married/partnered), (2) returned at the 2022 wave, and (3) had valid data provided by both partners in 2018 and 2022.

### Measures

#### Outcomes

*Anxiety symptoms* were measured using five items from the Beck Anxiety Inventory (Beck et al., 1988), which assesses the presence and frequency of anxiety symptoms within the last week. Responses ranged from 1 = Never to 4 = Most of the time. Anxiety symptoms were measured as a mean-score scale, such that higher values indicated greater anxiety symptoms (Smith et al., 2023). Values were set to missing if participants answered fewer than half of the items (α = 0.81 in 2018; α = 0.80 in 2022).

##### Perceived stress

A 10-item version of the validated Perceived Stress Scale (Cohen et al., 1983) was utilized to measure appraisal of stress in the last month, with responses ranging from 1 = Never to 5 = Very often. Perceived stress was measured as a mean-score scale, where higher values indicate greater perceived stress (Smith et al., 2023). Values were set to missing if participants answered fewer than half of the items (α = 0.86 in 2018; α = 0.84 in 2022).

##### Self-esteem

The 10-item Rosenberg Self-Esteem Scale (Rosenberg, 1965) was used to measure self-esteem. Participants were asked to respond to statements concerning feelings about self on a scale of 1 = Strongly agree to 4 = Strongly disagree, with the scale coded such that higher values indicate greater self-esteem (Smith et al., 2023). Self-esteem was measured as a mean-score scale, with values set to missing if participants answered fewer than half of the items (α = 0.87 in 2018; α = 0.86 in 2022).

#### Predictors

##### Everyday discrimination

A six-item version of the Everyday Discrimination Scale assessed how frequently participants experienced six distinct forms of discrimination (e.g., threatened, followed in stores) over the last 2 years, with response categories ranging from 1 = Almost every day to 6 = Never (Williams et al., 1997). Responses were coded such that higher values indicated greater perceived discrimination (Smith et al., 2023). Discrimination was measured as a mean-score scale, with values set to missing if participants answered fewer than half of the scale items (α = 0.80 in 2018).

##### Partner (e.g., vicarious) discrimination

Both partners were, independently of one another, asked to respond to the same 6 items concerning everyday discrimination. Thus, separate measures were included in models for individuals’ own and their intimate partners’ discrimination.

#### Covariates

All covariates were collected for each partner. Covariates included baseline values of each outcome, as well as the individuals’ own *age* (in years), *education* (less than HS [reference], HS degree, some college, college degree, education beyond college), *household wealth* (in quintiles), *employment status* (employed [reference], unemployed, retired, other), *race/ethnicity* (non-Hispanic White [reference], non-Hispanic Black, Hispanic, non-Hispanic other race), *marital support* (four-item scale), *marital strain* (three-item scale), *marital closeness* (1–4 scale), and *frequency of doing activities together with partner* (1–3 scale).

### Analytic strategy

Structural equation modeling (SEM) was used to estimate a series of lagged dependent variable (LDV) actor-partner interdependence model (APIM) equations. SEM allows for the simultaneous estimation of multiple regression equations, as well as for the covariance of all endogenous error terms. Moreover, SEM allows for the use of full information maximum likelihood (FIML) to address missing data. In this sample, 668 (90.00%) of 742 couples had valid data from both partners on all measures included in the analysis, and results from a model using listwise deletion produced the same significant results of interest as the models presented here (available upon request). In accordance with LDV modeling, the outcome measure at baseline was included as a predictor in each of the regression equations, such that coefficients can be interpreted in terms of *change* from 2018 to 2022. Overall, the model demonstrated adequate fit (RMSEA = 0.083; CFI = 0.954) according to established standards (Hu & Bentler, 1999). Models were estimated in wide format and stratified by gender, such that coefficients were estimated separately for men and women. Each set of models included adjustments for the individual’s own as well as their partner’s covariates, which are outlined above.

## Results

### Descriptive results

Descriptives are presented in Table 1 and, overall, indicate that, as expected, discrimination was relatively infrequent for both men and women, averaging approximately 1.5 on the 1–6 scale. However, men reported significantly more discrimination than women (*p* < .01). Additionally, anxiety symptoms and perceived stress were relatively low among both men and women, at both baseline and follow-up, averaging around 1.5 and 2.1 on the 1–4 and 1–5 scales, respectively. Self-esteem, on the other hand, was fairly high, averaging over 3.3 on the 1–4 scale for both men and women.

**Table 1:** Descriptive statistics, Health and Retirement Study (2018–2022), *N *= 1,484 partners from 742 couples).

Variables	Men (*n *= 742)		Women (*n *= 742)		Gender difference	Household
	*M* (*SD*)	%	*M* (*SD*)	%		
**Measures of interest**						
Discrimination (2018)	1.56 (0.68)		1.47 (0.59)		**	
Anxiety (2018)	1.45 (0.53)		1.49 (0.57)		–	
Anxiety (2022)	1.48 (0.52)		1.53 (0.57)		*	
Perceived stress (2018)	2.09 (0.64)		2.17 (0.68)		**	
Perceived stress (2022)	2.11 (0.64)		2.17 (0.64)		*	
Self-esteem (2018)	3.38 (0.47)		3.37 (0.49)		–	
Self-esteem (2022)	3.35 (0.45)		3.36 (0.49)		–	
**Covariates**						
Age (2018)	67.66 (9.21)		64.75 (8.88)		***	
Support (2018)	3.66 (0.46)		3.50 (0.61)		***	
Strain (2018)	1.84 (0.62)		1.88 (0.64)		–	
Closeness (2018; Very close)		70.78		61.31	***	
Time together (2018; A lot)		37.58		36.89	–	
Education					***	
Less than high school		10.24		9.57		
High school		40.84		47.44		
Some college		10.92		10.78		
College degree		21.83		19.41		
Beyond college		16.17		12.80		
Race/ethnicity					–	
Non-Hispanic White		73.05		74.12		
Non-Hispanic Black		10.65		9.84		
Non-Hispanic Other race		4.31		3.77		
Hispanic (any race)		6.87		6.60		
Employment (2018)					***	
Employed		38.24		34.95		
Unemployed		7.84		7.96		
Retired		52.97		46.42		
Other/Homemaker		0.95		10.66		
Wealth (2018; Household)					–	$692,032.70 ($2,034,052)

* *p* < .05.

** *p* < .01.

*** *p* < .001.

### Analytic results

The SEM results are presented in Table 2. For both men and women, individuals’ own discrimination was associated with more anxiety symptoms (*B = *0.11, *p < .*001 for men; *B = *0.07, *p < .*05 for women), greater stress (*B *= 0.14, *p* < .001 for men; *B = *0.15, *p < .*001 for women), and reduced self-esteem (*B* = −0.09, *p* < .001 for men; *B =* −0.07, *p < .*01 for women) at follow-up, accounting for baseline levels of the outcome. Additionally, models revealed only limited evidence of vicarious discrimination, where partner discrimination at baseline was associated with increased anxiety symptoms 4 years later, for men only (*B = *0.07, *p < .*05). This shows that husbands experienced increased anxiety symptoms over time when their wives experienced discrimination, but the same was not true for wives when their husbands experienced discrimination. However, post-hoc Wald tests revealed no significant gender difference in the coefficients for vicarious (i.e., partner’s) discrimination on follow-up anxiety.

**Table 2:** Results of dyadic lagged structural equation model concerning loneliness and cognition over 4 years (*N* = 742 opposite-gender couples).

Variables	Anxiety		Perceived stress		Self-esteem	
	Husbands (*n *= 742)	Wives (*n *= 742)	Husbands (*n *= 742)	Wives (*n *= 742)	Husbands (*n *= 742)	Wives (*n *= 742)
	*B* (*SE*)	*B* (*SE*)	*B* (*SE*)	*B* (*SE*)	*B* (*SE*)	*B* (*SE*)
**Measures of interest**						
Discrimination (own)	0.11*** (0.03)	0.07* (0.03)	0.14*** (0.03)	0.15*** (0.04)	−0.09*** (0.02)	−0.07** (0.02)
Discrimination (partner)	0.07* (0.03)	0.00 (0.03)	0.01 (0.03)	0.01 (0.03)	−0.02 (0.02)	0.01 (0.02)
Anxiety (own)	0.47*** (0.03)	0.51*** (0.03)	–	–	–	–
Perceived stress (own)	–	–	0.45*** (0.03)	0.42*** (0.03)	–	–
Self–esteem (own)	–	–	–	–	0.52*** (0.03)	0.57*** (0.03)
**Covariates**						
Age (own)	0.01** (0.00)	0.01 (0.00)	0.01 (0.00)	0.00 (0.00)	−0.01* (0.00)	−0.00 (0.00)
Age (partner)	−0.00 (0.00)	−0.00 (0.00)	0.00 (0.00)	0.00 (0.00)	−0.00 (0.00)	−0.00 (0.00)
Marital support (own)	0.01 (0.04)	−0.00 (0.04)	−0.09 (0.05)	−0.04 (0.04)	0.00 (0.03)	0.05 (0.03)
Marital support (partner)	−0.02 (0.03)	0.03 (0.05)	0.01 (0.04)	−0.08 (0.05)	0.04 (0.03)	−0.06 (0.04)
Marital strain (own)	−0.02 (0.03)	0.06 (0.03)	0.00 (0.04)	0.03 (0.04)	−0.00 (0.03)	−0.01 (0.03)
Marital strain (partner)	−0.03 (0.03)	0.02 (0.03)	0.03 (0.04)	0.02 (0.04)	−0.00 (0.03)	−0.08** (0.03)
Marital closeness: very close (own)^a^	0.02 (0.04)	−0.02 (0.04)	0.03 (0.05)	−0.08 (0.05)	−0.02 (0.03)	−0.01 (0.03)
Marital closeness: very close (partner)^a^	−0.04 (0.04)	0.05 (0.05)	0.01 (0.05)	0.13* (0.05)	0.01 (0.03)	0.01 (0.04)
Time together: a lot (own)^b^	0.02 (0.04)	0.05 (0.04)	0.01 (0.04)	−0.02 (0.04)	−0.02 (0.03)	−0.00 (0.03)
Time together: a lot (partner)^b^	0.02 (0.04)	−0.04 (0.04)	0.04 (0.04)	0.09* (0.04)	−0.01 (0.03)	−0.00 (0.03)
Education (own)^c^						
High school degree	−0.14* (0.06)	−0.05 (0.07)	−0.04 (0.07)	−0.03 (0.08)	0.09* (0.05)	−0.07 (0.05)
Some college	−0.16* (0.07)	−0.03 (0.08)	−0.14 (0.09)	−0.02 (0.09)	0.13* (0.06)	−0.07 (0.07)
College degree	−0.17* (0.07)	−0.08 (0.08)	−0.09 (0.08)	−0.11 (0.09)	0.12* (0.05)	−0.04 (0.06)
Beyond college	−0.09 (0.07)	−0.09 (0.08)	−0.08 (0.08)	−0.07 (0.09)	0.11 (0.06)	−0.03 (0.07)
Education (partner)^c^						
High school degree	−0.12* (0.06)	−0.06 (0.06)	−0.15* (0.07)	0.03 (0.07)	0.02 (0.05)	−0.01 (0.05)
Some college	−0.14 (0.08)	−0.05 (0.08)	−0.14 (0.09)	0.13 (0.09)	0.11 (0.06)	−0.03 (0.06)
College degree	−0.15* (0.07)	−0.06 (0.07)	−0.18* (0.09)	0.09 (0.08)	0.04 (0.06)	0.03 (0.06)
Beyond college	−0.13 (0.08)	−0.11 (0.08)	−0.07 (0.09)	0.03 (0.09)	0.06 (0.06)	0.03 (0.06)
Race/ethnicity (own)^d^						
Non–Hispanic Black	−0.06 (0.12)	−0.14 (0.13)	−0.32* (0.14)	−0.19 (0.14)	0.10 (0.09)	0.19 (0.10)
Non–Hispanic Other race	−0.08 (0.09)	0.18 (0.10)	−0.25* (0.10)	0.04 (0.11)	0.02 (0.07)	−0.00 (0.08)
Hispanic (any race)	0.07 (0.09)	0.09 (0.11)	0.01 (0.12)	−0.20 (0.12)	−0.08 (0.08)	0.09 (0.08)
Race/ethnicity (partner) ^d^						
Non–Hispanic Black	0.08 (0.12)	−0.01 (0.12)	0.15 (0.14)	0.10 (0.14)	−0.02 (0.09)	−0.06 (0.10)
Non–Hispanic Other race	0.07 (0.09)	−0.07 (0.09)	0.25* (0.11)	0.04 (0.11)	−0.10 (0.07)	0.01 (0.07)
Hispanic (any race)	−0.24* (0.10)	−0.02 (0.11)	−0.12 (0.12)	0.26* (0.12)	0.07 (0.08)	−0.19* (0.08)
Employment (own)^e^						
Unemployed	0.15* (0.06)	0.10 (0.07)	0.28*** (0.08)	0.23** (0.08)	−0.11* (0.05)	−0.14** (0.05)
Retired	−0.01 (0.04)	0.01 (0.05)	−0.01 (0.05)	0.08 (0.05)	0.05 (0.03)	−0.03 (0.04)
Other/Homemaker	−0.38* (0.16)	−0.04 (0.06)	−0.03 (0.19)	0.06 (0.07)	0.16 (0.12)	−0.04 (0.05)
Employment (partner)^e^						
Unemployed	0.01 (0.06)	−0.07 (0.07)	0.07 (0.08)	0.01 (0.08)	0.05 (0.05)	0.04 (0.05)
Retired	−0.01 (0.04)	0.05 (0.04)	0.00 (0.05)	−0.00 (0.05)	−0.01 (0.03)	0.06 (0.03)
Other/Homemaker	0.04 (0.05)	−0.06 (0.17)	0.00 (0.06)	0.07 (0.19)	0.06 (0.04)	−0.11 (0.13)
Wealth (household)^f^						
Quintile 2	−0.00 (0.05)	−0.02 (0.06)	−0.11 (0.06)	0.04 (0.06)	0.06 (0.04)	−0.07 (0.04)
Quintile 3	−0.03 (0.05)	−0.03 (0.06)	−0.03 (0.06)	−0.06 (0.06)	0.04 (0.04)	−0.00 (0.04)
Quintile 4	0.01 (0.05)	−0.11 (0.06)	−0.03 (0.07)	−0.08 (0.07)	0.12** (0.04)	−0.02 (0.05)
Quintile 5	−0.01 (0.06)	−0.07 (0.06)	0.10 (0.07)	−0.02 (0.07)	0.07 (0.05)	−0.02 (0.05)
**Model fit**						
CFI	0.954					
RMSEA	0.083					

a Reference: Not very close.

b Reference: Not a lot of time together.

c Reference: Less than high school degree.

d Reference: Non–Hispanic White.

e Reference: Employed.

f Reference: Quintile 1.

* *p* < .05.

** *p* < .01.

*** *p* < .001.

## Discussion

This brief report aimed to identify longitudinal dyadic associations of intimate partners’ experiences of everyday discrimination with anxiety symptoms, perceived stress, and self-esteem over a 4-year span in a sample of midlife and older couples. Overall, findings indicated that, although infrequent, discrimination was associated with poorer mental and emotional health for *individuals themselves* across all three outcomes, while the impact of vicarious discrimination on a *dyadic partner’s well-being* was only observed for men’s anxiety. We discuss the implications of these results for theory and future research concerning discrimination and dyadic health.

First, results concerning individuals’ mental and emotional health following exposure to discrimination cohere with research establishing discrimination as a chronic stressor that can produce stress and anxiety, and undermine self-esteem (e.g., Ayalon & Gum, 2011; Earnshaw et al., 2016; Mouzon et al., 2017; Stokes, 2019; Williams et al., 1997). This holds true for those with intimate partners, despite the social and emotional support available through these relationships. Indeed, although effect sizes were modest, they were comparable to effect sizes of perceived discrimination on mental health among the broader aging population (e.g., Stokes & Moorman, 2020). Although older adults report less perceived discrimination than younger counterparts, any level of exposure poses risks to their psychological well-being, suggesting that discrimination is critical to study in older populations, including married/partnered populations.

Second, results concerning implications of vicarious discrimination for intimate partners were more complex. Neither self-esteem nor perceived stress were affected by partner discrimination over the 4-year study period, contrary to our expectations. Yet, partner discrimination *did* lead to more anxiety symptoms after 4 years, albeit only for husbands. This suggests that not all aspects of well-being are equally impacted by a partner’s discrimination. More research is needed to identify reasons why partners’ anxiety *was* susceptible to dyadic influence following discrimination exposure, yet one potential explanation lies in “emotional contagion” (Hatfield et al., 1993; Stokes, 2017). Previous research has indicated that anxiety can “spread” between partners, such that having an anxious partner is a risk factor for experiencing greater anxiety oneself (Stokes, 2017). Thus, discrimination exposure may provoke anxiety symptoms in the affected individual, which in turn leads to greater anxiety for their partner. Whether perceived stress and/or self-esteem are likewise “contagious” among older couples has yet to be evaluated. It is worth noting, as well, that, whereas anxiety and stress both demonstrated significant changes over the 4-year study period (see Table 1), self-esteem was consistent for both men and women across timepoints, suggesting that it may be more stable and trait-like and less susceptible to the influence of psychosocial stressors such as perceived everyday discrimination.

Interestingly, only men’s anxiety symptoms were affected by their female partner’s discrimination exposure, and not vice versa. This gendered finding was not anticipated by the literature review or theoretical framework utilized, yet it is consistent with a growing literature suggesting that wives more strongly affect husbands’ mental health than the reverse (e.g., Jessee & Stokes, 2026; Thomeer et al., 2013) and raises the need for further research on the interpersonal and social processes at play following stress exposure among aging couples. We would note, however, that this partner effect did not *differ significantly* by gender, and that the gender difference demonstrated by the results is merely suggestive.

### Limitations

There are a number of limitations to the present study that are worth noting. First, the 2018–2022 study frame allows for a longitudinal analysis of the most recent waves of HRS LBQ data, but attrition between waves was likely impacted by the COVID-19 pandemic in 2020. Moreover, response rates to the LBQ have been lower in recent waves than in the past, potentially biasing the analytic sample. Attrition—whether on the basis of death, deteriorating health, or divorce and relationship dissolution—may result in a sample that overrepresents the healthiest individuals and relationships, perhaps underestimating true associations of interest in the population. An additional limitation in the data is that experiences of everyday discrimination, perceived stress, anxiety, and self-esteem were asked using different time scales (e.g., “in the past two weeks,” “over the past month”), which complicates the interpretation of these measures as all reflecting “current” or “contemporaneous” values. Finally, the population of same-gender couples in the present data set was too small for analysis, limiting our sample to mixed-gender couples. Future work should focus on differences—as well as similarities—among LGBTQ+ older couples’ experiences of discrimination and mental health.

Despite these limitations, this research makes an important contribution to the literature on discrimination exposure and dyadic health science. Although the mental and emotional repercussions of discrimination were clearer for the affected individuals themselves than for their intimate partners, results indicated that men whose female partners were exposed to discrimination experienced more anxiety symptoms over the 4-year study period. Our findings—including null dyadic results—suggest that the implications of dyadic discrimination for mental health may be more nuanced than anticipated, and may be more pertinent for certain aspects of mental and emotional well-being than for others. Future work should seek to determine which aspects of mental health are most—and least—susceptible to partner influences, in order to devise translational interventions that are appropriately tailored (e.g., couple-level vs. individual-level interventions), depending on the outcome of interest.

## Data Availability

HRS data are made available to researchers at https://hrs.isr.umich.edu. Analytic files are available from the authors upon request.
